# The Experience of a Single NHS England Trust on the Impact of the COVID-19 Pandemic on Junior and Middle-Grade Doctors: What Is Next?

**DOI:** 10.3390/ijerph181910413

**Published:** 2021-10-03

**Authors:** Antonios Revythis, Sidrah Shah, Synthia Enyioma, Aruni Ghose, Meenash Patel, Afroditi Karathanasi, Elisabet Sanchez, Stergios Boussios

**Affiliations:** 1 Department of Medical Oncology, Medway NHS Foundation Trust, Windmill Road, Gillingham, Kent ME7 5NY, UK; antonios.revythis@nhs.net (A.R.); sidrah.shah@nhs.net (S.S.); synthia.enyioma1@nhs.net (S.E.); aruni.ghose@nhs.net (A.G.); meenash.patel@nhs.net (M.P.); a.karathanasi@nhs.net (A.K.); elizabet.sanchez@nhs.net (E.S.); 2 Faculty of Life Sciences & Medicine, School of Cancer & Pharmaceutical Sciences, King’s College London, London SE1 9RT, UK; 3 AELIA Organization, 9th Km Thessaloniki-Thermi, 57001 Thessaloniki, Greece

**Keywords:** COVID-19, Corona, coronavirus, SARS-CoV-2, burnout, medical staff, health personnel

## Abstract

The COVID-19 pandemic has undoubtedly affected all national healthcare systems at different levels. In countries heavily hit by the pandemic, it was reported that healthcare workers were asked to work long hours, had increased workload, were faced with difficult decisions, and that the resources were stretched. As such, the COVID-19 pandemic would create the perfect storm for burnout in healthcare workers. Within this context, we conducted a survey in a district general hospital in Southeast England. We focused on doctors in training, in different specialties. This survey included parts of the Maslach Burnout Inventory for healthcare professionals, along with other relevant questions, such as the financial impact and seeking of psychological support. The results showed moderate levels of emotional exhaustion, but high levels of personal satisfaction, a positive impact on doctors finances and very low levels of seeking support.

## 1. Introduction 

The National Health Service (NHS), which is the government-funded healthcare system of the United Kingdom (U.K.), is the world’s fifth largest employer of doctors [1]. The 2018 nationwide British Medical Association (BMA) survey revealed that approximately 80% of doctors in the U.K.—mostly junior and middle-grade doctors—were considered high risk for burnout as evidenced by overwhelming exhaustion, depersonalization, and reduced personal efficiency. In the same study, one-third of the above population was diagnosed with a mental health condition and reported usage of alcohol, drugs, and self-medication as coping mechanisms. Similar findings were reported in medical students in the U.K. [2]. Burnout has been associated with depression and substance abuse [3,4]. This includes high levels of stress or mental health difficulties, but the stigma of reporting these intimidates and deters doctors from seeking help [5]. 

Since 2020, the COVID-19 pandemic has affected approximately 150 million people and taken over 3 million lives [6]. Furthermore, it has posed an unprecedented challenge to the healthcare profession worldwide and increased the existing risk of burnout for doctors [7]. Similar to studies conducted in China and Italy, the BMA reported that more than 50% of doctors have had progressive worsening of general health and well-being since the first wave [8]. Chatterjee et al. observed 90% of junior doctors to be affected by psychiatric morbidities such as depression, stress, and anxiety disorders [9]. This is further worsened by social factors: moving out of homes to reduce risk of transmission or restricting contact with vulnerable family [9]. Furthermore, emergency COVID-19 rotas and redeployment, unclear treatment guidelines or escalation plans, reduced training opportunities, and increased patient mortality have precipitated burnout in an already overstretched workforce [10].

We conducted a survey in our district general hospital in Southeast England among the junior and middle-grade doctors to investigate the impact of COVID-19 pandemic on the work/life balance of the participants, the potential physical or mental burnout, and the financial impact. We also focused on whether resilience has been developed during the pandemic through relative changes between the first and second waves of the pandemic.

## 2. Methods

### 2.1. Design and Sample 

We used survey data from 61 anonymised questionnaires to junior and middle-grade doctors in a medium-sized district general NHS hospital in Kent (U.K.) serving a population of 400,000. The survey included 38 questions involving demographics, work-related circumstances (including specialty and field of work) and 22 items from the Maslach Burnout Inventory (MBI) for medical personnel (MBI-HSS), the most widely used burnout assessment tool [11]. The questionnaire was e-mailed to 220 trainees in total. Only physicians practicing at the trust were included. Consultant-grade doctors were not included. This was because, generally, consultant-level doctors in the U.K. are able to organise their work schedule differently to their junior and middle-grade counterparts and tend not to be onsite overnight. Therefore, the essence of the questionnaire and the results may have been skewed. Statistical analysis was performed using IBM SPSS Statistics 27 (Microsoft Windows 32-bit) software. Descriptive statistics are presented as mean ± standard deviation for continuous variables, and absolute count/percentage for categorical variables.

### 2.2. Measures

The MBI is an assessment tool used to measure burnout defined in three sub-categories: emotional exhaustion (9 questions), depersonalisation (5 questions), and personal accomplishment (8 questions) (see Appendix A). Every question in these subsections has a response range from never, rarely, sometimes, frequently and always; to assist with quantification, we assigned scores ranging from 0 to 4. Additional questions were added. On the question regarding the current work/life balance, the scores ranged from very dissatisfied to somewhat dissatisfied, neither satisfied nor dissatisfied, somewhat satisfied and very satisfied. The participants were asked to grade how busy their workload was with responses of light, normal, or busy. When the participants were asked to comment on how frequently they feel burned out, the responses provided were never, rarely, sometimes, often, or always. MBI profiles were identified using the MBI Z-scores and MBI mean scores. The profile types are Engaged, Ineffective, Overextended, Disengaged, and Burnout [11].

The project was registered within the Audit and Quality Improvement as per hospital policy. Ethical approval was not required for this project. Cronbach’s alpha was used to measure the internal consistency of the MBI questionnaire, per component. Mean and standard deviation were calculated using Excel MS.

## 3. Results

**Figure 1:**
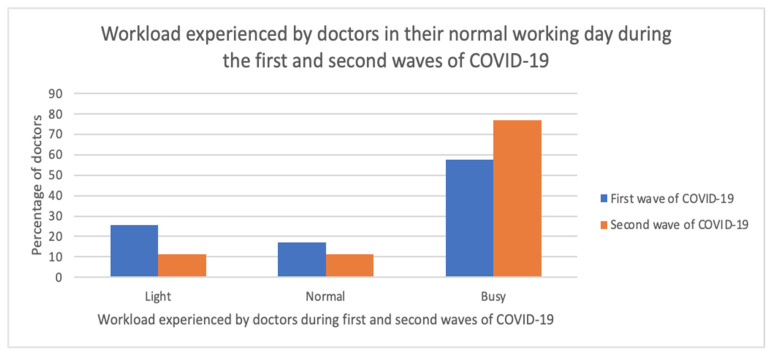
Workload experienced by doctors in their normal working day during the first and second waves of COVID-19.

**Figure 2:**
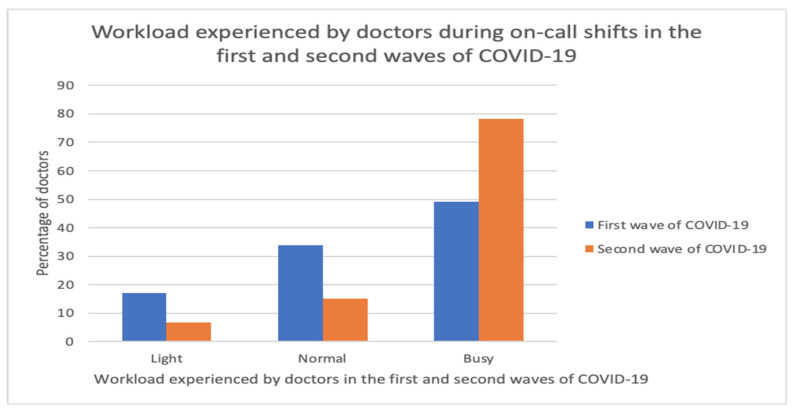
Workload experienced by doctors during their on-call shifts in the first and second waves of COVID-19.

Sixty-one junior and middle-grade doctors agreed to complete the questionnaire. The participants’ demographics are summarised in Table 1. This included all grades of junior and middle-grade doctors: foundation year 1 (F1), foundation year 2 (F2), core trainees (CT), internal medical trainees (IMT), acute care common stem (ACCS), and specialty years one to seven (ST1-7) and Medical Training Initiative (MTI) trainees. The vast majority of the participants (93.44%, 57/61) agreed that COVID-19 has changed their workload (Table 2, Figure 1 and Figure 2). More specifically, the majority of the participants suggested that workload increased during both waves of the pandemic. In the first wave, busy workloads were reported in 34/61 and 29/61 in normal working days and on-call shifts, respectively. These numbers increased during the second wave of the pandemic to 47/61 (78.3%) in normal working days and on-call shifts alike. When the participants were asked to grade their work/life balance, 46 out of 61 reported feeling very or somewhat dissatisfied at the time of the survey (Table 2). About half of the participants were redeployed (34/61), as reported in Table 3. A small proportion of 6/61 were redeployed in both waves of the pandemic. 

**Scheme 1:**
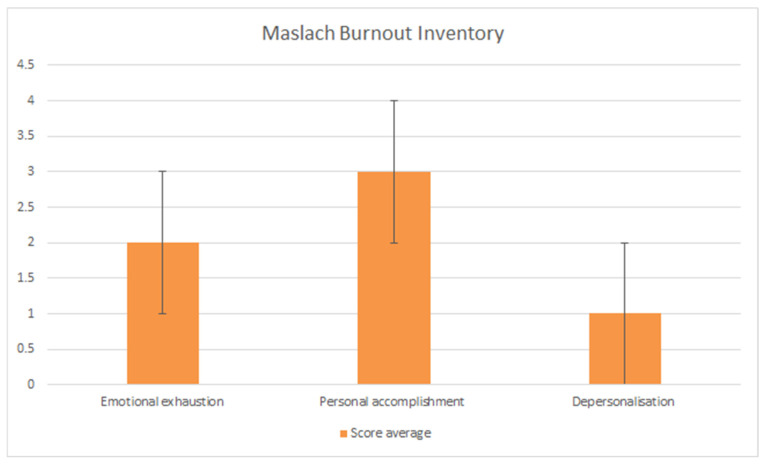
Graphic representation of the mean of the three Maslach Burnout Inventory results with standard deviation.

When the participants were asked if they would answer the questions regarding emotional exhaustion, depersonalisation, and personal accomplishment, 35/61 (57.3%) identified that their responses would be more favourable before the second wave. We did not find any difference between the different training grades. The mean of Maslach Inventory score was calculated with standard deviation (Scheme 1) for the three categories: with a mean emotional exhaustion of two, personal accomplishment of three, and depersonalisation of one out of four-point scores. On analysis, Cronbach’s alpha, a measure of internal reliability, was satisfactory for two out of the three components of the MBI questionnaire (0.90 in emotional exhaustion concept, 0.77 for personal accomplishment and 0.67 fordepersonalization, likely due to the low number of questions) [12].

When the participants were asked regarding financial impact, 27/61 (44.2%) noted a positive impact and 17/61 (27.8%) identified a negative impact. The remainder (17/61, 27.8%) did not identify an impact to their finance.

At the end of the questionnaire, participants were asked if they asked for help or if they sought advice over their feelings; 52/61 (88.1%) did not seek any help or advice.

## 4. Discussion 

This survey aimed to explore the impact of the COVID-19 pandemic on junior and middle-grade doctors at an NHS district general hospital in the U.K. through the first and second waves. We focused on the increased workload, physical and mental burnout, financial impact, and the lack of clear communication from doctors to seek help. Though prevalent in the first wave, these were more prominent in the second wave of the pandemic and the effects still persisted in between the first and second waves with no clear return to normality. Since the outbreak of the COVID-19 pandemic, healthcare professionals have faced a variety of challenges worldwide [13]. Our results are consistent with the challenges documented in the literature. These include higher workload with longer working hours and frequent on-call shifts, high levels of anxiety and psychological distress—one cause of this could be shortages of personal protective equipment (PPE)—and subsequent overall burnout [14,15,16,17]. 

The General Medical Council (GMC) report of medical education and practice in the U.K. found that workload was already demanding for doctors before the pandemic. In 2019, 69% of doctors were working beyond their rostered hours regularly and 28% felt they could not cope with their workload at least one day a week [18]. This has shifted since the onset of the pandemic. The majority of our respondents (56/61, 93.44%) felt their workload changed due to the COVID-19 pandemic (Figure 1 and Figure 2). This is similar to the existing literature, where excessive workloads and working hours can negatively impact work/life balance and cause inadequate rest and support, emotional exhaustion, and burnout [19,20]. During the first wave, 34/59 doctors (57.63%) felt that their normal working day was busy. This is in contrast to the second wave where 47/61 (77.05%) felt they were busy in their normal working day. A similar pattern was observed for on-call shifts with doctors reporting the second wave was busier. The increased workload in the second wave may be attributed to the rapidly transmissible COVID-19 variant B.1.1.7, which dominated the U.K. and caused surges in hospitalisations and deaths [21]. Furthermore, 1 in 50 people were testing positive during the peak of the second wave and NHS hospitals had over 34,000 COVID-19 patients in England—80% higher than the peak of the first wave [21]. The higher rate of COVID-19 patients and hospitalisations would explain why the second wave felt much busier than the first.

Workload was demonstrated to have increased due to junior and middle-grade doctors’ treating more COVID-19 patients and adapting to changing COVID-19 protocols, mandatory use of PPE before seeing patients, longer working hours with no compensation for working beyond rostered hours, and rota changes to incorporate more on-call shifts [18,22,23,24]. Hoonakker et al. found that on-call shifts, such as night shifts, have a higher workload than day shifts; regular shifts longer than 12 h are also associated with higher levels of burnout compared to those lasting 8 h [25,26]. However, there are conflicting studies on workload of doctors working in surgery. Non-urgent elective operations have been cancelled in order to support the wider COVID-19 response and surgical staff have been redeployed throughout the hospital [27,28]. Though this may appear to have primarily reduced the workload of surgeons, the changing work environment has caused worry for surgeons due to delay in surgical services, fewer training opportunities, and redeployment to critical care or medicine [27,29,30]. Our results showed mixed responses from those working in surgery—many commented that their workload was light during the first wave but busy in the second wave, with the majority having normal workloads in between the waves. The majority of our respondents (33/61, 54.10%) were working in medicine compared to 11/61 (18.03%) working in surgery and only 2/61 (3.28%) in critical care. With workload significant for intensive care unit (ICU) staff along with the greatest COVID-19 exposure, perhaps our results are skewed as the majority of our respondents worked in medicine [31]. Further research would be useful to compare workloads in between specialties in more detail. 

Workload for junior and middle-grade doctors may have increased due to redeployment to manage the increased demand from COVID-19 patients [13,28]. For doctors redeployed to intensive treatment units (ITUs), workload was high, with management of multiple unwell patients, demanding rotas, and filling in for absenteeism or unwell staff during the pandemic [32,33]. This can be related to medical specialties also managing high numbers of COVID-19 patients. Redeployment has also been found to contribute to burnout [28,34]. With redeployment, some doctors were working in and adapting to unfamiliar areas without their usual support network or regular debriefings [28]. Furthermore, redeployment may have led to higher exposure to COVID-19 patients, which can lead to prolonged periods of time wearing protective equipment [35]. In essence, redeployment would be a result of needing to meet excessive demand and it is understandable that doctors may feel overwhelmed with this [31,35]. Gemine et al. found that concerns arising from redeployment were due to the lack of choice or the uncertainty of moving departments, exacerbated by poor communication of these decisions [36]. For these reasons, future research should focus on the impact of redeployment on morale and burnout in junior and middle grade doctors in further detail.

The 2018 nationwide BMA survey found junior and middle-grade doctors to be at the highest risk of burnout. The study used the Oldenburg Burnout Inventory (OLBI) and revealed that burnout was driven more by exhaustion rather than depersonalisation from one’s role as a doctor [1]. Our study used the MBI and we found similar results, with reduced personal achievement and emotional exhaustion scoring the highest. Interestingly, there was no marked difference between roles. Of 61 participants, 45 (73.7%) were significantly dissatisfied with their work/life balance, whereas 35/61 (57.3%) were frequently exhausted from their work. Equal numbers of participants (35/61, 57.3%) identified that their responses to questions involving individual burnout components would be more favourable before the second wave. This reiterates how the second wave was more demanding.

In addition to burnout, we recorded subjective measures of stress. More than three-quarters of the workforce surveyed reported feeling heightened stress levels at work during the COVID-19 pandemic, but fewer than one in four of those with stress had accessed workplace support. The main reason reported was that support was not required, despite feeling heightened stress levels. This phenomenon is not unusual among healthcare workers, with a delay between event-timing and reflective practice that allows staff to acknowledge the emotional impact of their work [34]. As not all of those recognising their need for support sought help, this suggests that the structure through which support is being offered may not be meeting the needs of those it is intended to serve. Inadequate time and not wanting colleagues to know they were seeking support were two of the main barriers to access. Digital platforms are effective in delivering psychological treatment, and therefore can be offered by organizations and accessed discreetly at an appropriate time for the individual [34]. One of the few factors found to be protective against burnout, depression, and anxiety was feeling supported at work. Peer supervision and strong teamwork were previously shown to strengthen workplace well-being [36]. Healthcare employers could support service leads, ward managers, senior nurses, and local supervisors in understanding their critical role in supporting their staff to prevent the development of burnout.

While caring for the unwell remains a central aspect of why individuals enter the profession, it is essential to understand the concept of monetary value when working up close and living through the pandemic to see how this offsets the cost of an eventual burnout. When the participants were asked regarding a financial impact, 27/61 (44.2%) noted a positive effect and 17/61 (27.8%) identified a negative impact. The remainder, 17/61 (27.8%), did not determine an impact on their finance. Increased working hours, if outside of the contract and as locum at escalated rates, would lead to higher income. However, the intensity of these hours is further reflected, with 56% and 48% of respondents mentioning that during the first wave, shifts were busier both within regular working hours and on-calls, respectively. Of 61 participants, 56 said yes to working in “hot” environments as a matter of standard placement or redeployment at any one time over the course of both waves. A lack of correct PPE meant some individuals opted to pay for their own for better protection.

Our study had its share of limitations. First, it was a single-centre survey. We hope to expand this to a multi-centre study in the future. Second, we had a small cohort of 61 participants out of all the junior and middle-grade doctors in our centre. Third, eager participation was proportional to the level of dissatisfaction. Those who had been affected tremendously were eager to fill out the questionnaire. The rest were not interested. Fourth, our cohort did not include consultant response, which can be assessed in the future. Fifth, most of our respondents were from medicine and hence there was no equal distribution among specialties.

## 5. Conclusions

Our survey was targeted to explore the psychological impact of the COVID-19 pandemic on junior and middle-grade doctors. Our results were notable of moderate levels of emotional exhaustion, low levels of depersonalisation, and high levels of personal achievement. These results, coupled with the positive financial impact by the majority of doctors and the increased workload, create an environment of mixed positive and negative consequences. This probably indicates the resilience of junior and middle-grade doctors, who, despite the increased workload and adverse effects of the pandemic, reported high levels of personal achievement. Another key issue highlighted in our survey was that junior and middle-grade doctors were reluctant to seek advice or support. We think that the findings of our survey are a starting point of understanding how the COVID-19 pandemic has affected medical personnel, suggesting that further insight into the protective factors, coping mechanisms, and differences between groups of doctors should be sought, providing potential future directions.

## Figures and Tables

**Table 1:** Characteristic of the participants completing the survey.

Age Group (Years)	Participants (*n* = 61)
20–29	31
30–39	22
Over 40	6
Prefer not to say	2
**Relationship status**	
Single	23
In a relationship	19
Married	18
Prefer not to answer	1
**Level of training**	
F1	17
F2	13
SHO in training (CT, IMT, ACCS, ST1-ST2)	13
SpR in training (ST3 and above)	9
SHO staff grade	5
MTI and Specialty Doctors	4

**Table 2:** Workload and work-life changes due to COVID-19.

Has Workload Changed due to COVID-19?	Participants (*n* = 61)
No	4
Yes	57
**How busy were the normal days during the 1st wave of the pandemic?**	
Busy	34
Light	15
Normal	10
Skipped	2
**How busy were the on-call shifts during the 1st wave of the pandemic?**	
Busy	29
Light	10
Normal	20
Skipped	2
**Level of satisfaction/dissatisfaction on current work/life balance**	
Very or somewhat dissatisfied	46
Neither satisfied nor dissatisfied	11
Very or somewhat satisfied	5

**Table 3:** COVID-19 working experience of the survey participants.

Working during the COVID-19 First Wave	Participants (*n* = 61)
No	10
Yes	51
**Working in “hot’’ environments during second COVID-19 wave**	
No	5
Yes	56
**Have you been redeployed?**	
No	27
Yes—both waves	6
Yes—during the first wave	16
Yes—during the second wave	12

## Appendix A

**Demographics/Personal Information**	
Are you	Male
	Female
	Non-binary
	Prefer not to say
Are you	20–30 years old
	30–40 years old
	Over 40 years old
	Prefer not to say
What describes your marital status?	Single
	In a relationship
	Married
	Separated/Divorced
	Prefer not to answer
Has the pandemic had an impact on your finances?	Yes, Positive
	Yes, Negative
	No
What best describes your role?	Foundation year 1
	Foundation year 2
	SHO in training (CT, IMT, ACCS, ST1-ST2)
	SHO staff grade
	MTI
	SpR in training
	Specialty doctor/ SAS
Working circumstances	
What specialty do you currently work in?	Medicine
	Critical Care
	Emergency Medicine
	Surgery
	Other (please specify)
Were you working during the first wave of COVID-19 (March 2020–June 2020)?	Yes
	No
Which hospital were you working at during the first wave of COVID-19?	Current Hospital
	Other (please specify)
During the second wave of the pandemic (November 2020–current), have you worked in a COVID-19 “hot” environment (managing suspected or confirmed cases)?	Yes
	No
Have you been redeployed during the first and second waves?	Yes—During the first wave
	Yes—During the second wave
	Yes-Both waves
	No
Do your feel that the workload has changed as a result of COVID-19?	Yes
	No
How busy do you feel your normal working days have been (during the first wave, between the waves, during the second wave)?	Light
	Normal
	Busy
How busy do you feel your on-call shifts have been (during the first wave, between the waves, during the second wave)?	Light
	Normal
	Busy
Please indicate your current level of satisfaction or dissatisfaction regarding your current work-life balance	Very dissatisfied
	Somewhat dissatisfied
	Neither satisfied/not dissatisfied
	Somewhat satisfied
	Very satisfied
Please indicate how often these statements are true for you	
I feel emotionally drained from work	Never
	Rarely
	Sometimes
	Frequently
	Always
I feel used up at the end of the workday	Never
	Rarely
	Sometimes
	Frequently
	Always
I feel fatigued when I get up in the morning and have to face another day on the job	Never
	Rarely
	Sometimes
	Frequently
	Always
Working with people all day is really a strain for me	Never
	Rarely
	Sometimes
	Frequently
	Always
I feel burned out from my work	Never
	Rarely
	Sometimes
	Frequently
	Always
I feel frustrated by my job?	Never
	Rarely
	Sometimes
	Frequently
	Always
I feel I’m working too hard on my job	Never
	Rarely
	Sometimes
	Frequently
	Always
Working with people directly puts too much stress on me	Never
	Rarely
	Sometimes
	Frequently
	Always
I feel like I am at the end of my rope	Never
	Rarely
	Sometimes
	Frequently
	Always
I can easily understand how my patients feel about things	Never
	Rarely
	Sometimes
	Frequently
	Always
I deal very effectively with the problems of my patients?	Never
	Rarely
	Sometimes
	Frequently
	Always
I feel I am positively influencing other people’s lives through my work	Never
	Rarely
	Sometimes
	Frequently
	Always
I feel very energetic	Never
	Rarely
	Sometimes
	Frequently
	Always
I can easily create a relaxed atmosphere with my patients	Never
	Rarely
	Sometimes
	Frequently
	Always
I feel exhilarated after working closely with my patients	Never
	Rarely
	Sometimes
	Frequently
	Always
I have accomplished many worthwhile things in this job	Never
	Rarely
	Sometimes
	Frequently
	Always
In my work, I deal with emotional problems very calmly	Never
	Rarely
	Sometimes
	Frequently
	Always
I feel I treat some patients as if they are impersonal objects	Never
	Rarely
	Sometimes
	Frequently
	Always
I have become more callous toward people since I took this job	Never
	Rarely
	Sometimes
	Frequently
	Always
I worry that this job is hardening me emotionally	Never
	Rarely
	Sometimes
	Frequently
	Always
I do not really care what happens to some patients	Never
	Rarely
	Sometimes
	Frequently
	Always
I feel patients blame me for some of their problems	Never
	Rarely
	Sometimes
	Frequently
	Always
Would you answer the above questions more favourably before the second wave?	Yes
	No
Have you sought help of advice about your feelings?	No
	Yes. If yes by whom?
